# Preoperative anxiety management in pediatric patients: a systemic review and meta-analysis of randomized controlled trials on the efficacy of distraction techniques

**DOI:** 10.3389/fped.2024.1353508

**Published:** 2024-02-19

**Authors:** Muhammad Saqlain Mustafa, Muhammad Ashir Shafique, Syeda Dua E Zehra Zaidi, Amna Qamber, Burhanuddin Sohail Rangwala, Aftab Ahmed, Syeda Mahrukh Fatima Zaidi, Hussain Sohail Rangwala, Muhammad Musab Nafees Uddin, Mirha Ali, Mohammad Arham Siddiq, Abdul Haseeb

**Affiliations:** ^1^ Department of Medicine, Jinnah Sindh Medical University, Karachi, Pakistan; ^2^ Department of Paediatrics, National Institute of Child Health, Karachi, Pakistan; ^3^ Department of Medicine, Dow University of Health Sciences, Karachi, Pakistan

**Keywords:** pediatric preoperative anxiety, distraction techniques, nonpharmacological interventions, pediatric surgery, systemic review and meta-analysis

## Abstract

**Background:**

This study addresses the pervasive issue of heightened preoperative anxiety in healthcare, particularly among pediatric patients. Recognizing the various sources of anxiety, we explored both pharmacological and nonpharmacological interventions. Focusing on distraction techniques, including active and passive forms, our meta-analysis aimed to provide comprehensive insights into their impact on preoperative anxiety in pediatric patients.

**Methods:**

Following the PRISMA and Cochrane guidelines, this meta-analysis and systematic review assessed the efficacy of pharmaceutical and distraction interventions in reducing pain and anxiety in pediatric surgery. This study was registered on PROSPERO (CRD42023449979).

**Results:**

This meta-analysis, comprising 45 studies, investigated pharmaceutical interventions and distraction tactics in pediatric surgery. Risk of bias assessment revealed undisclosed risks in performance and detection bias. Distraction interventions significantly reduced preoperative anxiety compared to control groups, with notable heterogeneity. Comparison with Midazolam favored distraction techniques. Subgroup analysis highlighted varied efficacies among distraction methods, with a notable reduction in anxiety levels. Sensitivity analysis indicated stable results. However, publication bias was observed, suggesting a potential reporting bias.

**Conclusion:**

Our study confirms distraction techniques as safe and effective for reducing pediatric preoperative anxiety, offering a valuable alternative to pharmacological interventions.

**Systematic Review Registration:**

https://www.crd.york.ac.uk/prospero/display_record.php?RecordID=449979, PROSPERO [CRD42023449979].

## Background

Numerous healthcare professionals and patients share concerns regarding the heightened anxiety that individuals often endure while gearing up for various medical procedures. This pervasive issue not only poses a threat to patients' mental and physical well-being, but also correlates with undesirable outcomes, such as an increased requirement for anesthesia, elevated risks of surgical complications, and suboptimal rehabilitation (1).

Individuals undergoing surgical procedures often experience heightened anxiety stemming from myriad factors. This emotional distress may arise from fear of mortality, apprehensions about regaining consciousness after anesthesia, uncertainty surrounding the procedure, concerns about potential pain, loss of control over the situation, feelings of isolation, and the emotional strain of being separated from loved ones. Notably, statistics indicate that 65%–80% of pediatric patients experience preoperative anxiety, underscoring the prevalence and significance of this issue in the healthcare landscape (2).

The manifestation of anxiety in pediatric patients undergoing medical procedures is diverse and can be observed through various behavioral and physiological indicators. In more severe cases, young children might unexpectedly urinate, display hypertonic behavior, or attempt to escape from medical staff (3).

Among the various stages in the preoperative period, the induction of anesthesia is notably distressing for pediatric patients, evident in both their behavioral and physiological responses. This pivotal moment introduces distinct challenges for anesthesiologists and surgeons, as the stress experienced by both patients and parents becomes palpable. Compounded by heightened anxiety, there is a noteworthy occurrence of last-minute refusals from pediatric patients, a manifestation of preoperative fear that adds a layer of complexity to the anesthesia and surgical processes (4).Therefore, acknowledging and effectively addressing preoperative anxiety in pediatric patients is important. The use of sedative drugs has emerged as a valuable strategy to mitigate anxiety before surgery, aiding in smoother separation from friends and family and reducing discomfort during the induction process (5). However, it is essential to recognize that some young patients may resist taking medications, and the efficacy of drugs is not guaranteed. In some cases, medications may not produce the intended calming effects, leading to unforeseen side effects, such as irritability and disinhibition (6).

Furthermore, additional drawbacks associated with the use of sedative drugs in pediatric patients include prescription costs, safety concerns (including the risk of airway blockage or respiratory depression in the absence of vigilant monitoring), increased demands on nursing personnel and additional supplies, and potential for delayed hospital discharge (7). Consequently, there is growing interest in the use of nonpharmacological treatments. Medical practitioners and parents often turn to distraction as a non-pharmacological technique to alleviate the pain and anxiety associated with medical procedures in pediatric patients (8).

The reduction of pain, anguish, and anxiety is attributed to diverting the attention of juvenile patients towards pleasant stimuli, thereby impeding the processing of painful sensations. Distraction treatments come in various forms and are broadly categorized as active or passive. Passive forms involve activities such as watching a film or listening to music where the patient receives external stimuli. On the other hand, active distraction entails direct engagement, with young patients participating actively, often under the guidance of an adult. Activities such as painting, playing with toys, and using virtual reality fall into the realm of active distraction, providing diverse and interactive avenues for alleviating distress during medical procedures (6).

To comprehensively evaluate the impact of distraction on preoperative anxiety, specifically in pediatric patients, we conducted a meta-analysis incorporating studies with a larger sample size. Our objective was to provide a robust foundation for therapeutic practices by scrutinizing the effects of both active and passive modes of distraction on preoperative anxiety in this demographic. This nuanced analysis aims to provide valuable insights for tailoring interventions that address the unique needs of pediatric patients undergoing medical procedures.

## Method

### Protocol

Using data from a prior registration on PROSPERO (CRD42023449979), we performed a thorough meta-analysis and systematic review to evaluate the efficacy of pharmaceutical interventions and distraction tactics in lowering pain and anxiety in children undergoing surgery. The PRISMA and Cochrane Handbook of Systematic Review and Intervention guidelines were followed in our investigation.

### Search strategy

We searched electronic databases such as PubMed, MEDLINE, Embase, and Scopus for relevant literature. Using pertinent keywords like “Child,” “Pediatric,” “preschool,” “preoperative management,” “anxiety,” and “anxiety management,” our search returned publications up until June 2023. Supplementary File 1 contains a separate version of the full-mesh phrase. The language used for the data search was only English.

### Eligibility criteria

We included research that satisfied certain requirements: individuals under the age of 18 who had any kind of surgery, whether minor or major, any method of distraction utilized during a minor or large medical treatment, such as virtual reality, video games, psychological preparation, entertainment videos, books, music, clown intervention, guided tour, and smartphone and tablet, and a control group receiving any pharmacological anxiolytic medication during the surgical procedure. Preoperative anxiety and anxiety levels across various distraction tactics and a pharmacological control group were assessed as primary and secondary outcomes. Cohort studies and randomized controlled trials (RCTs) were the only types considered. Studies in non-English languages, those involving non-human subjects, those involving adults older than 18, studies without the desired results, and study types other than randomized controlled trials (RCTs) or cohorts (e.g., case-control, case series, editorials, single-arm studies) were among the studies that we excluded.

### Study screening and data extraction

Two reviewers independently reviewed the articles. After articles that met the inclusion criteria were first included, they were later excluded based on the full-text review, title, and abstract. A third reviewer arbitrated any disputes or disagreements by reaching consensus. Details about the authors, year of publication, baseline characteristics (population age, follow-up, sample size), type of distraction technique used in the intervention, duration, type of pharmacological anxiolytics used in the control group, and outcome data (preoperative anxiety and anxiety level) were all taken from pertinent studies.

### Data analysis and risk of bias

We used the Cochrane Collaboration's RevMan version 5.4 and Comprehensive Meta-Analysis version 3.3 to analyze the data. Risk ratios (RR) with a 95% confidence interval were utilized, along with random effect model. This model for preoperative anxiety and anxiety level was used to construct funnel plots for the primary and secondary outcomes. The heterogeneity between the included studies was evaluated using the I₂ statistic. *I*_2_ over 50% was considered significant heterogeneity, and the sensitivity analysis was interpreted considering the study's characteristics. Using the Cochrane methodology for the risk of bias, the risk of bias in the included RCTs was evaluated. Studies pertaining to selection, reporting, other, and performance biases were categorized as low, high, or unknown risk. Preoperative anxiety and anxiety level outcomes of eligible studies were analyzed using a funnel plot for publication bias.

## Results

### Study selection and characteristics

**Figure 1:**
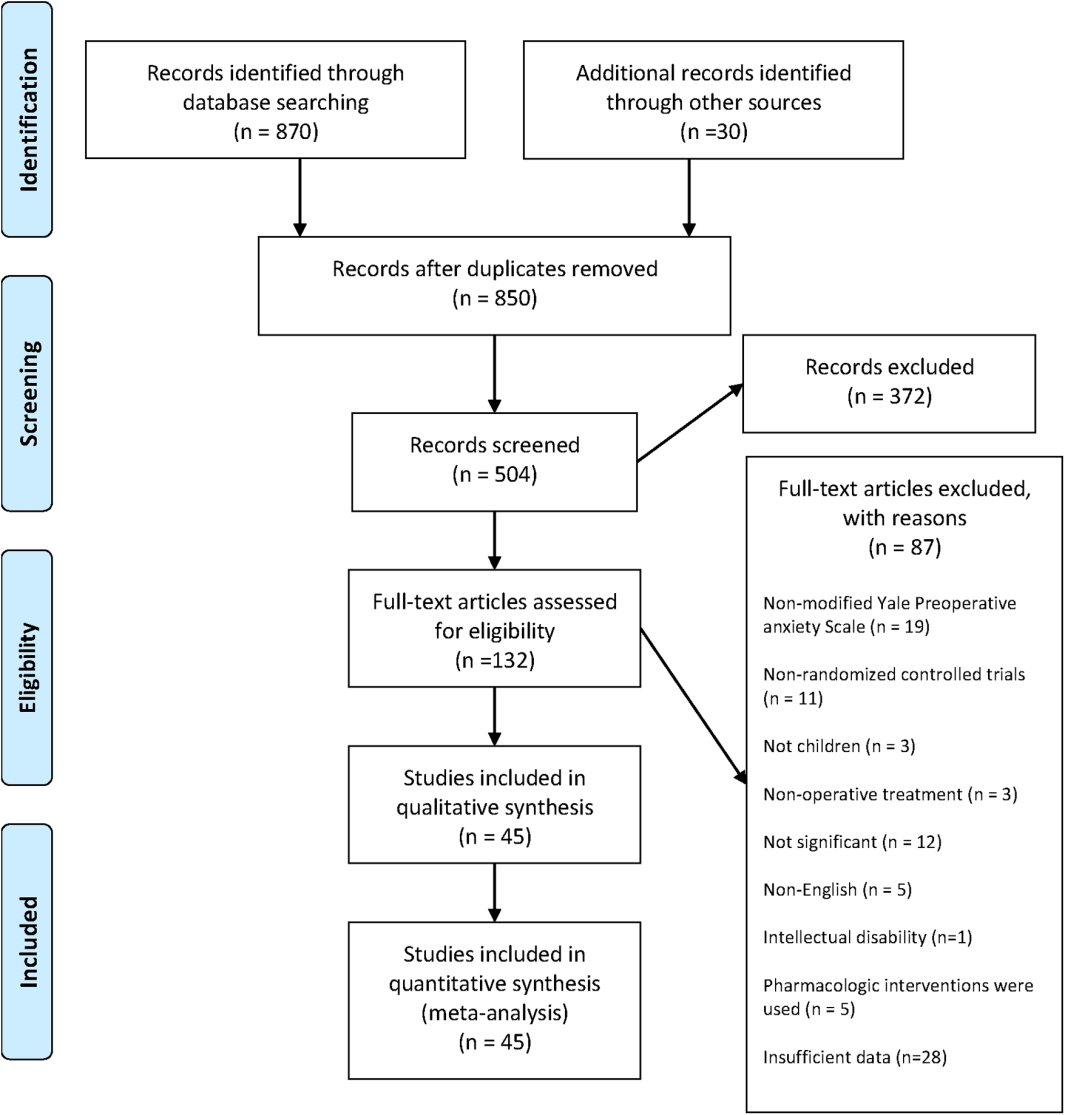
The PRISMA flowchart.

Depicted is the schematic representation of our search and selection process, encompassing data until July 2023. The initial pool of 850 studies underwent rigorous curation, resulting in 504 unique records after duplicates were removed. Subsequent scrutiny of titles and abstracts led to the exclusion of 372 irrelevant studies, followed by a thorough assessment of the full text of 132 articles. Of these, 87 were excluded for specific reasons. This meticulous process culminated in the final selection of 45 studies that met the eligibility criteria. The PRISMA Flowchart presented in Figure 1 lists the studies that met our inclusion requirements. The baseline characteristics of the patients are shown in Table 1.

**Table 1:** Characteristics of studies included in the systematic review.

Study	Year	Country	Intervention setting	Method of intervention	Intervention period		Control group	Mean age T/C	Sex male/female	Outcome measures
					Holding area	Induction room				
William Li et al. (9)	2007	Honkong	Outpatient	Therapeutic play	NA	NA	Routine care	7–12	0/63	C-SAS
Yu et al. (10)	2009	China	inpatient	Music	NA	NA	Routine care	8.26 ± 2.83/7.87 ± 3.35	36	m-YPAS score
Golan et al. (11)	2009	Occupied	Inpatient	Clown Intervention	YES	YES	Routine care	4.5	NA	m-YPAS
Kim et al. (12)	2010	Korea	inpatient	Maternal recorded voice	YES	NA	Routine care	8 ± 42/82 ± 42	1510/13	m-YPAS
Vangoli et al. (13)	2010	Italy	Inpatient	Clown	YES	YES	Routine care	6.85/7.3	T70/30 C75/25	m-YPAS
Huet et al. (14)	2011	France	Outpatient	Hypnosis	NA	NA	Routine care	8 (5–12)/9 (5–12)	15/15	m-YPAS
Cuzzocoria et al. (15)	2013	Italy	Inpatient	Psychological preparation activities	YES	YES	Routine care	NA	42/8	m-YPAS
Tunney et al. (16)	2013	Northern Ireland	Outpatient	Story book	NA	NA	Routine care	7.7	0/44 (55%F)	HFRS
Lim et al. (17)	2013	Singapore	NA	Head Play	YES	YES	Routine care	9.4/8.77	T27/3 C24/6	m-YPAS
Karimi et al. (18)	2014	Iran	Inpatient	Orientated tour of Operation Room	NA	YES	Routine care	NA	25/45	m-YPAS
Heilbrunn et al. (19)	2014	USA	Inpatient	Hospital Clown	YES	YES	Routine care	7.70/7.48	36/36	m-YPAS
Gao et al. (20)	2014	China	Inpatient	Game	YES	YES	Routine care	4.52/4.37	T20/9 C23/7	m-YPAS
He et al. (21)	2015	China	NA	Therapeutic play	NA	NA	Routine care	16/15.66	61/34	SAS-C scores
Batuman et al. (22)	2016	Turkey	Outpatient	Preoperative information video	NA	YES	Routine care	NA	NA	m-YPAS
Bumin Aydin et al. (23)	2016	Turkey	Inpatient	Play Dough	YES	NA	Routine care	5 (3–7)	51/53	m-YPAS
Fortier et al. (24)	2016	USA	Outpatient	Web-Based Tailored Intervention	YES	YES	Routine care	4.3 ± 1.8/c4.4 ± 1.7	46	m-YPAS score
Cumino et al. (25)	2017	Brazil	Inpatient	Smartphone and information leaflet	YES	YES	Routine care	5.29/5.65	59/25	m-YPAS
Hatipoglu et al. (26)	2018	Turkey	Outpatient	Video	NA	YES	Routine care	7.6/7.6	33/33	m-YPAS
Ryu et al. (27)	2018	Korea	Outpatient	Virtual reality tour of the operating theatre	YES	YES	Routine care	6 (5–8)	50/30	m-YPAS
Wantanakorn et al. (28)	2018	Thailand	Outpatient	Mobile Application	NA	NA	Routine care	9.22 6 3.43/9.57 6 3.06	35/25	m-YPAS
Liu et al. (29)	2018	China	Inpatient	Children's ride-on car	YES	YES	Midazolam/Routine care	3.6/3.4	T19/15 C17/17	m-YPAS
Meletti et al. (30)	2019	Ireland	Outpatient	Psychological preparation	NA	YES	Basic preparation, 33% O2 and 66% N2O	NA	87/13	modified Yale Preoperative Anxiety Scale (m-YPAS)
Vangoli et al. (31)	2019	Italy	Inpatient	Relaxation-guided imagery	NA	YES	Basic prep	8.3+−1.4	32/28	m-YPAS, FLACC
Churasia et al. (32)	2019	India	NA	Video game	NA	YES	Routine care	5.3/5.2	T34/6 C31/9	m-YPAS
Park et al. (33)	2019	Korea	Inpatient	Virtual reality tour of the operating theatre	YES	YES	Routine care	85 (69–101),81 (64–105)	47/33	m-YPAS
Stewart et al. (8)	2019	USA	Inpatient	Tablet-based interactive distraction	YES	YES	Midazolam	6.8 (2.4) y (4–12 y);6.9 (2.5) y (4–12 y)	59/43	m-YPAS
Khulman et al. (34)	2020	Netharland	Inpatient	Music Therapy	YES	YES	NA	mi[6.9 (3.3–11.1)months]	164/15	COMFORT-Behavior scale,
Park et al. (35)	2020	Korea	Inpatient	Wagon transportation	YES		Normal stretcher	4.3/4.3	36/40	m-YPAS
Seyedhejazi et al. (36)	2020	Iran	Inpatient	Reading booklet prepared by paediatric anesthesiologist	NA	NA	Midazolam	9.12 ± 1.72;8.45 ± 1.86	25/23	State-Trait Anxiety Inventory for Children
Jung et al. (37)	2021	USA	Inpatient	Virtual Reality	YES	YES	NA	8.0+−2.3	NA	m-YPAS
Wang et al. (38)	2021	China	Inpatient	Video Distraction	NA	NA	Routine care	NA	NA	m-YPAS
Uyar et al. (39)	2021	Turkey	Outpatient	Tablet			Midazolam			m-YPAS
			Outpatient	Video Game			Midazolam			m-YPAS
Cordray et al. (40)	2021		NA	Books	NA	YES	Basic Prep	7.64 + −2.2	82/62	m-YPAS
Jin et al. (41)	2021	China	Inpatient	Self-produced audio-visual animation	YES	YES	Routine care		53/47	m-YPAS
Huang et al. (42)	2021	China	Inpatient	Music	YES	YES	Routine care	7.53/6.3	50/38	m-YPAS
Li et al. (43)	2021	China	Inpatient	Situational adaptation training combined with childlike nursing	NA	NA	Routine care	8 ± 6	NA	VAS score
Bumin Aydin et al. (44)	2021	Turkey	Inpatient	Story book	YES	YES	Routine care	c 6.39 ± 0.6 6.53 exp ± 0.8	53/49	m-YPAS
Wu et al. (45)	2022		Inpatient	Virtual Reality	YES	YES	Basic prep	NA	86/13	m-YPAS
Sahin et al. (46)	2022	Turkey	Inpatient	Tablet/Computer	YES	YES	Midazolam	NA		m-YPAS
Yang et al. (47)	2022	China	Outpatient	Animated picture book	YES	YES	Routine care	4.7/4.4	43/73	m-YPAS
Castany et al. (48)	2023		Inpatient	Virtual tour of operating tour	YES	NA	Basic prep	NA	95/30	m-YPAS
Luo et al. (49)	2023	China	Outpatient	Virtual Realty	NA	YES	Routine care			m-YPAS
Levay et al. (50)	2023	USA	Outpatient	Active distraction (tablet)	NA	NA	Midazolam	3–5 years	60/39	m-YPAS
Chambon et al. (51)	2023	France	Inpatient	Electric Toy Car	YES	YES	Routine care	4.7/6.3	24	mYPAS-SF
Wang et al. (52)	2023	China	Inpatient	Music	YES	YES	Routine care	7.63/6.97	86	m-YPAS
			Inpatient	Cartoon/Animation	YES	YES	Routine care	7.63/7.23	89	m-YPAS

NA, not available; T, test group; C, control group; CI, confidence interval; m-YPAS, modified Yale Preoperative Anxiety Scale; app, application; VR, virtual reality; VAS Score, Visual Analogue Scale; SAS-C, Separation anxiety scale for children; FLACC, Face, Legs, Activity, Cry and Consolability; C-SAS, Chemotherapy Symptom Assessment Scale; HFRS, Hemorrhagic fever with renal syndrome.

### Risk of bias

While conducting a comprehensive review, it was noted that a significant proportion of the studies included in our analysis had undisclosed risks of performance and detection bias as these critical factors were not explicitly addressed in the text. However, the level of selection bias was generally low to moderate in these studies. Notably, randomized trials displayed variability in reporting randomization procedures, with the majority lacking participant masking to the interventions, as anticipated in the experimental design.

Despite these observations, overall management of other potential latent biases was effective, leading to a relatively consistent risk of bias across trials. Supplementary Figure S1 offers a detailed overview of each trial's potential biases, providing valuable insights into the study landscape.

### The outcomes between the distraction intervention group and the control group at the entrance of the operation room

**Figure 2:**
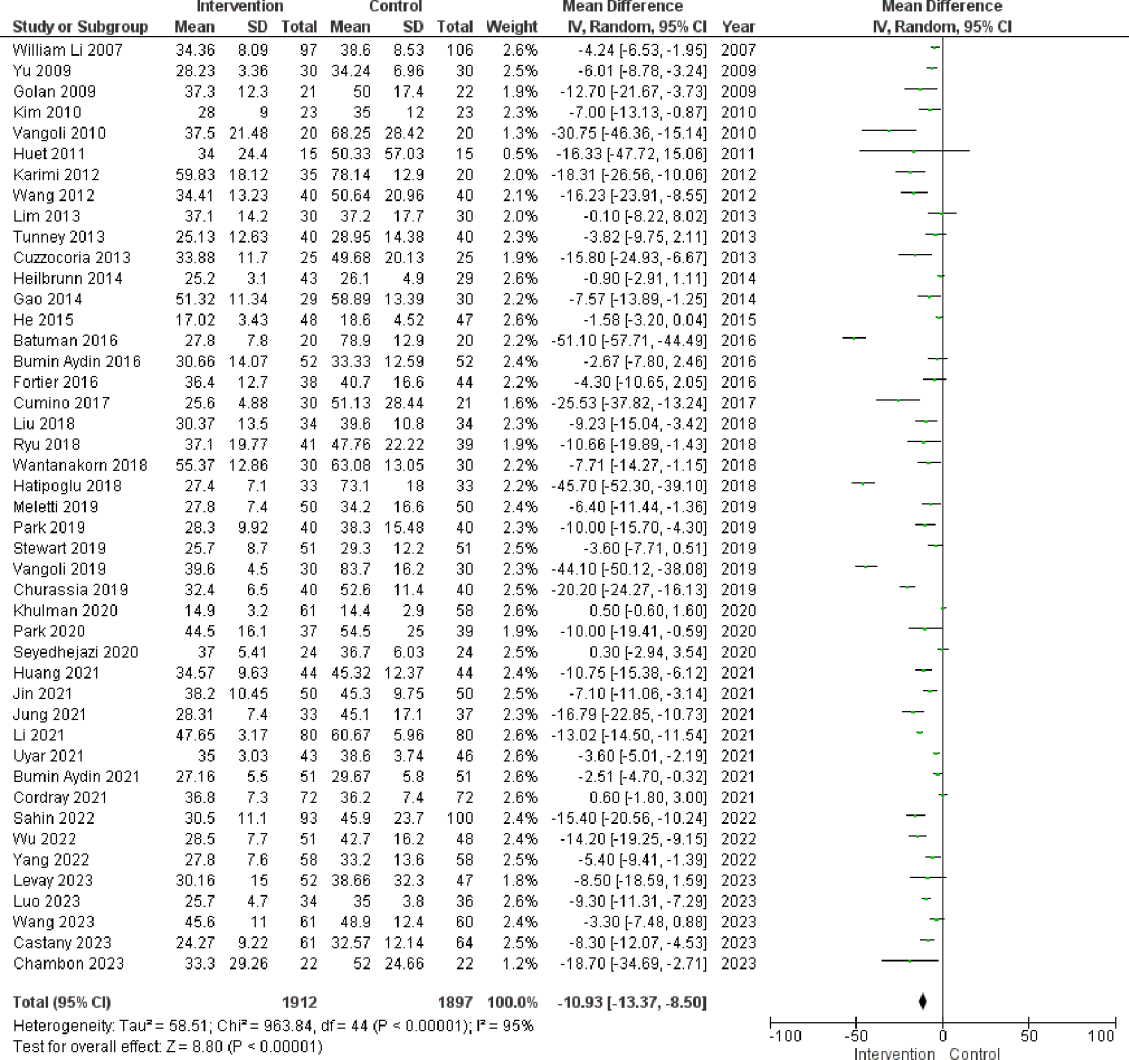
Illustrates the influence of distraction interventions on preoperative anxiety in children undergoing surgery. The figure compares the outcomes between the Intervention group and the control group at the entrance of the Operation Room.

The combined results from the studies suggest a noteworthy reduction in anxiety levels by 10.93 in the distraction group compared to the control group (with a mean difference of −10.93 and a 95% confidence interval of −13.37 to −8.50),as shown in Figure 2. However, the *Q*-test and *I*_2_ statistics revealed substantial heterogeneity among the included studies (*P* < 0.00001, *I*_2_ = 95%).

### Comparison with midazolam

**Figure 3:**
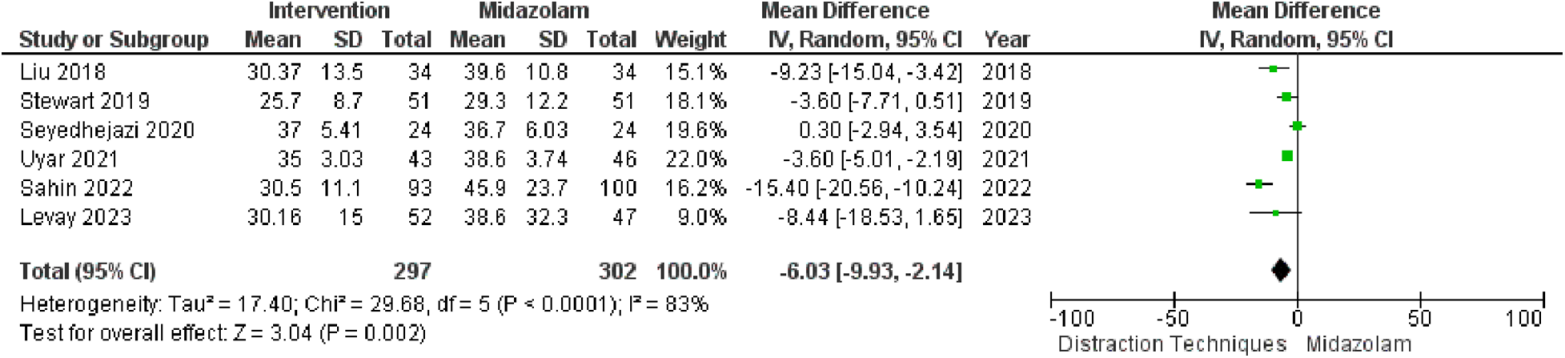
Distraction technique vs. midazolam use on preoperative anxiety in children undergoing surgery.

In the context of preoperative anxiety control, Figure 3 highlights a limited number of studies that have compared the utilization of midazolam and distraction techniques. The collective analysis of these studies demonstrated that distraction techniques offer greater efficacy in managing pre-operative anxiety, as indicated by a risk ratio (RR) of −6.03 within a 95% confidence interval (CI) ranging from −9.93 to −2.14 (*P*-value <0.002). Notably, a substantial degree of heterogeneity was observed with an I2 value of 83%.

### Subgroup analyses

**Figure 4:**
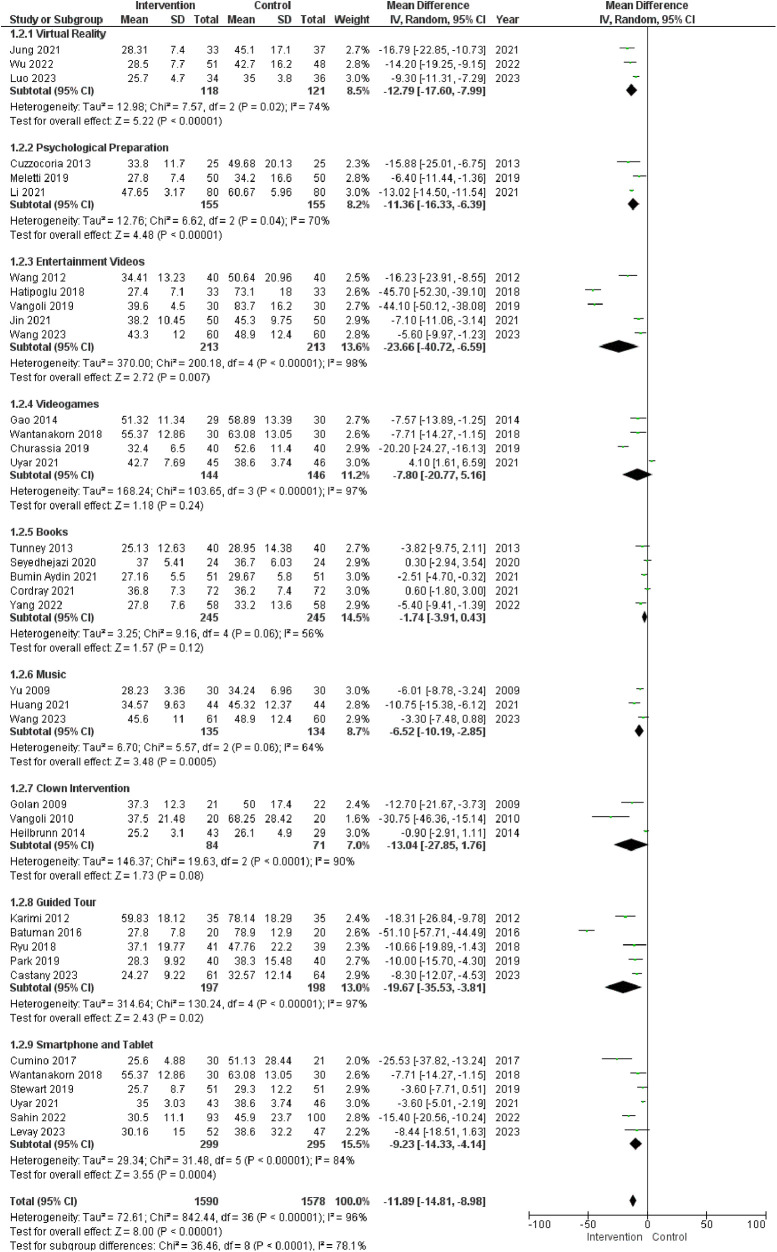
Subgroup analysis of different distraction techniques. The figure compares the outcomes between the Intervention group and the control group at the entrance of the Operation Room.

Analysis of the data presented in the table, which examines the effectiveness of both active and passive distraction methods, reveals significant differences in anxiety reduction between the distraction and control groups. These results underscore the superior efficacy of distraction interventions in lowering anxiety levels compared to the control group. Notably, the most substantial anxiety reduction was observed in the “Entertainment Videos” subgroup (MD = −23.66, 95% CI: −40.72 to −6.59) (*P* < 0.007), demonstrating a statistically significant difference. Similarly, the “Virtual Reality” (MD = −12.79, 95% CI: −17.60 to −7.99) (*P* < 0.0001) and “Psychological Preparation” (MD = −11.36, 95% CI: −16.33 to −6.39) (*P* < 0.0001) subgroups exhibited significant reductions. On the other hand, the “Videogames” subgroup (MD = −7.80, 95% CI: −20.77 to 5.16) (*P* = 0.24) did not show a statistically significant difference in anxiety reduction. Similarly, the “Books” (MD = −1.74, 95% CI: −3.91 to 0.43) (*P* = 0.12) and “Clown Intervention” (MD = −13.04, 95% CI: −27.85 to 1.76) (*P* = 0.08) subgroups were also non-significant. In contrast, the “Music” subgroup (MD = −6.52, 95% CI: −10.19 to −2.85) (*P* = 0.0005), “Guided Tour” (MD = −19.67, 95% CI: −35.53 to −3.81) (*P* = 0.02), and “Smartphone and Tablet” (MD = −9.23, 95% CI: −14.33 to −4.14) (*P* = 0.0004) exhibited statistically significant anxiety reduction. The overall effect size across all subgroups was also noteworthy (MD = −11.89, 95% CI: −14.81 to −8.98) (*P* < 0.0001). However, itItit is important to note that even within these subgroups, substantial heterogeneity persisted. Figure 4.

### Sensitivity analysis

**Figure 5:**
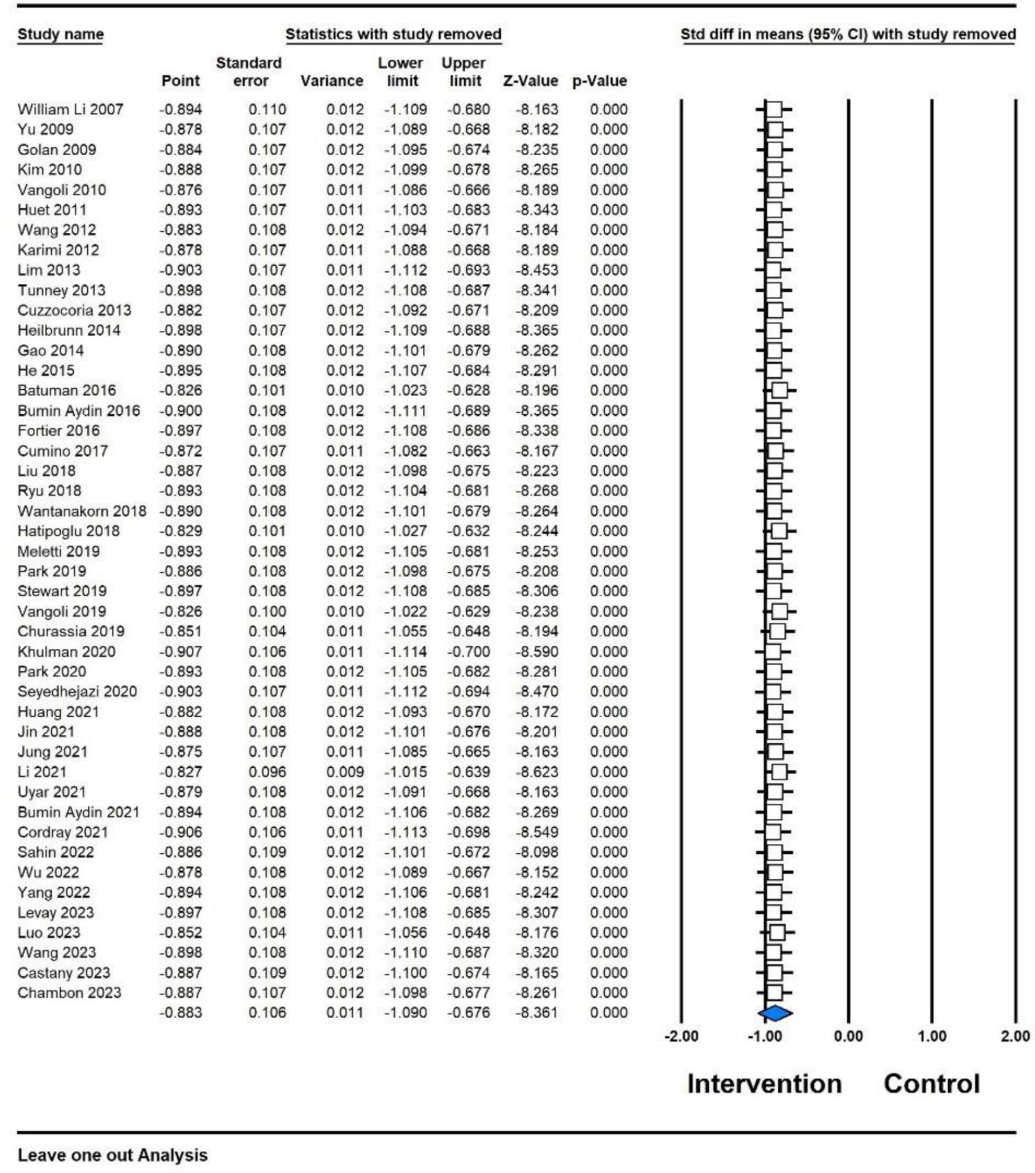
Sensitivity analysis of figure 1—intervention (distraction) and control.

To assess the robustness of the results obtained in this meta-analysis, a sensitivity analysis was conducted using leave-one-out method (Figure 5). The purpose was to ascertain whether the pooled effect size remained consistent or underwent significant changes. The outcomes of this analysis provided insights into the stability of the meta-analysis results and the potential influence of individual studies on the overall findings. Sensitivity analysis indicated that the removal of any single study did not lead to substantial alterations in the pooled effect size. This observation implies that the conclusions drawn from the meta-analysis are relatively stable and that the overall results demonstrating a more favorable effect of distraction on preoperative anxiety compared to the control groups remain unchanged.

### Publication bias test

The funnel chart representation revealed a notable bias, with an aggregation of studies predominantly concentrated in the upper region of the triangular space. On visual assessment, the funnel plot revealed asymmetry and the begg test was significant (*z* = 3.77; *P* = 0.00008). Sensitivity analysis using the trim-and-fill method was performed with 11 imputed studies, which produced a symmetrical funnel plot. This pattern suggests the possibility of publication bias or outcome reporting bias, wherein studies exhibiting significant or favorable outcomes are more likely to be published or included in the analysis (Supplementary File 1).

## Discussion

Young children, from preschoolers to school-age, often struggle to grasp the need for and importance of surgery. This, along with being away from family in a new place with the fear of pain, creates various levels of stress and anxiety. While we relied solely on medicine for this purpose in the past, distraction techniques are increasingly being used to alleviate feelings of intense distress and fear in children undergoing surgical procedures (53). This study is an update on a previous meta-analysis performed by Wu et al. (54). Our analysis of 45 studies showed that the use of non-pharmacological distraction techniques serves as an effective means to alleviate distress and anxiety in pediatric patients preoperatively.

A group of diverse and complex neuropathophysiological mechanisms underlies the development of anxiety and the natural response to fear (55). Studies have demonstrated that preoperative anxiety is associated with increased anesthesia and analgesic requirements, adverse postoperative outcomes, and delayed recovery (1). Distraction and nonpharmacological techniques can directly or indirectly interfere with these underlying mechanisms and provide a simple and readily available method for reducing anxiety. Furthermore, a systematic review corroborated that nonpharmacological interventions can effectively reduce stress and anxiety levels in children, which was confirmed by the measurement of salivary cortisol levels (56).

The non-pharmacological distraction techniques explored in our study was Virtual reality, Psychological Preparation, Entertainment Videos, Videogames, Books, Music, Clown Intervention, Guided Tour, and Smartphone and Tablet.

Video distraction was a prevalent distraction tool applied across numerous studies that included cartoons (52, 57), relaxation-guided imagery (13), and self-produced audiovisual presentations (41). They were found to be effective tools for diverting children's attention to painful stimuli and curbing preoperative anxiety. Studies have shown that cartoons and audiovisual presentations are superior to traditional toy play (57) and only auditory interventions (26). Virtual reality (VR) interventions have been demonstrated to be an effective distraction technique owing to a high degree of immersion due to its improved tracking, stereoscopy, and wide field of view that can make Virtual reality experiences feel realistic and effectively divert the patients' attention from adverse stimuli (58). In a related review, Tas et al. validated the effectiveness of Virtual reality techniques in reducing preoperative anxiety and pain in various medical procedures (59). Accordingly, the use of smartphones and tablet-based interactive interventions has enabled pediatric patients to be more comfortable and has alleviated feelings of distress and anxiety. Nonetheless, balancing the benefits of distraction with the potential negative effects of prolonged screen time is important. However, in our study, video games did not have a substantial effect on preoperative anxiety, which may be due to factors such as effectiveness depending on the child's age and individual preferences. Therefore, more robust ongoing research is required to provide more specific efficacy rates for these techniques, catering to narrower age groups, and modified based on specific case characteristics.

Furthermore, psychological preparation provides a potent non-pharmacological method for managing preoperative anxiety in pediatric patients. These methods improve children's emotional health, cooperation during surgery, and overall recovery experiences by providing them with knowledge, coping skills, and a sense of control. Children can be given the tools they need to manage their anxiety in a healthy way using techniques such as progressive muscle relaxation, deep breathing, and guided imagery. Coping skills such as visualization, distraction tactics, and positive self-talk can help them deal with stress better. In addition, age-appropriate explanations, tours of the operating room, and introductions to medical staff could also create a comfortable environment and occupy the preoperative time period in a more relaxed way. In an experimental study, Kain et al. used a preoperative preparatory program that included role-playing, a hospital tour, and perioperative information (60). The program was conducted over a period of one to seven days and was adjusted based on age. They discovered that patients aged 6 years and up experienced the least anxiety if the program started more than 5–7 days prior to surgery (*P* < 0.04), emphasizing the crucial nature of psychological preparation (61). Parental involvement in the planning process can also create a supportive atmosphere.

Among the included studies, many other distraction methods were applied, such as music, clown-based intervention, and books. Music intervention has been reported to significantly reduce preoperative anxiety, as in our study, offering a cost-effective and non-invasive approach for alleviating perioperative psychological pressure (62, 63). Listening to music decreases the sympathetic nervous activity and activates the parasympathetic nervous system. Its widespread availability makes it particularly suitable for clinical use, especially in operating rooms, effectively lowering anxiety levels in children before anesthesia induction, consistent with prior meta-analysis results (9).

According to a study, when compared to the control group, the music group's postoperative patient satisfaction increased while the postoperative State-Trait Anxiety Inventory form 1 (STAI-1) score decreased after listening to their favorite music prior to elective inguinal hernia surgery, signifying its importance in anxiety controlling (64). Furthermore, In light of the conflicting findings in multiple studies about the impact of music therapy on blood pressure and heart rate, Agüero-Millan et al. propose that patients should select the music they listen to in order to enhance these physiological parameters, urging the need for more confounding studies to be conducted on this distraction technique (65).

Despite the statistical insignificance in our study, clown-based intervention has previously been shown to reduce preoperative anxiety in comparison to premedication (11). According to Vangoli (2010), clown interventions may create a more positive and memorable experience for children, potentially making future inductions less frightening than the amnesic effect of medication (13). However, it is important to consider the potential negative impacts that can occur in children with a fear of clowns (11). Despite statistical insignificance in our study, clown-based intervention has previously been shown to reduce preoperative anxiety in comparison to premedication (11). According to Vangoli (2010), clown interventions may create a more positive and memorable experience for children, potentially making future inductions less frightening compared to the amnesic effect of medication (13). However, it's important to consider the potential negative impact can occur in children with a fear of clowns (11). Factors that may have led to the insignificant correlation may be fear or discomfort, age appropriateness, cultural sensitivity, or a lack of understanding of such forms of entertainment.

In the context of utilizing books as a distraction technique, our meta-analysis did not reveal any statistical significance, but individual studies illuminate the promising benefits of employing diverse forms of books in pediatric preoperative settings. Notably, random factors, such as small sample sizes, methodological variances, and different individual preferences in terms of distraction, may have affected the results. This highlights the need for more in-depth trials to be conducted in the future, focusing on each non-pharmacological treatment for anxiety management in pediatric patients preoperatively and encompassing different age groups in the pediatric field.

In operative settings, midazolam is often used as an adjunct to reduce anxiety in pediatric patients, and our comparison demonstrated that distraction techniques are equally or more effective than midazolam. It is clinically significant, as midazolam is associated with prolonged onset of effects and adverse impacts such as agitation and restlessness. According to studies, the administration of midazolam is associated with an extended duration of hospitalization among patients (8, 66). Seiden et al. compared tablet-based interactive distraction to midazolam and found distraction to be superior in decreasing parental perception of anxiety (66).

### Limitations

While our study provides valuable insights into various distraction techniques aimed at easing preoperative anxiety in pediatric settings, it is crucial to acknowledge certain limitations. The diverse range of distraction methods explored is beneficial, yet there is still room for future research to uncover additional interventions that are not covered in our analysis. Despite our efforts to provide the most up-to-date insights until July 2023, the limited availability of comprehensive studies in the existing database prevents us from drawing broad conclusions. The diverse nature of pediatric patients, including factors such as age, sex, parental anxiety, and experience with pain, could influence the effectiveness of distraction techniques. Unfortunately, due to limitations in the design of the included studies, we could not conduct detailed analyses considering these factors. Additionally, the high heterogeneity observed among the studies, despite our efforts to account for it, suggests variations in how the studies were conducted and in the characteristics of the participants. Finally, the possibility of publication bias raises some concerns. Our study, while making strides in understanding distraction interventions, may be influenced by a bias towards publishing studies with significant or favorable outcomes. Despite our attempts to address this bias, it is important to interpret the results cautiously and acknowledge the potential impact of selective reporting on our findings.

## Conclusion

In summary, our study established distraction techniques as safe, inexpensive, and efficient methods for alleviating preoperative anxiety in the pediatric population, approving it as an alternative to pharmacologic interventions. However, additional research is required to compare various active and passive techniques to enhance our understanding of the intricate mechanisms underlying these techniques and to provide insights into their potential applications in different clinical scenarios.

## Data Availability

The original contributions presented in the study are included in the article/Supplementary Material, further inquiries can be directed to the corresponding author.
