# Supplementary material for: Preoperative anxiety management in pediatric patients: a systemic review and meta-analysis of randomized controlled trials on the efficacy of distraction techniques
Source: Front Pediatr. 2024 Feb 19;12:1353508. doi: 10.3389/fped.2024.1353508 (PMC10909818; doi:10.3389/fped.2024.1353508)
Supplement: Supplementary file 1 [file Datasheet1.pdf]

Supplementary Material

Search Strategy:

(“child”[MeSH Terms] OR “child” [All Fields] OR “children” [All Fields] OR “pediatrics” [All Fields] OR “pediatric” [All Fields] OR “preschool” [All Fields]) AND (“preoperative”[All Fields] OR “preoperative management” [All Fields] OR “perioperative” [All Fields]) AND (“anxiety”[MeSH Terms] OR “anxious” [All Fields] OR “anxiety management” [All Fields]) AND (“randomized controlled trial”[Publication Type] OR “randomized” [Title/Abstract] OR “placebo” [Title/Abstract])

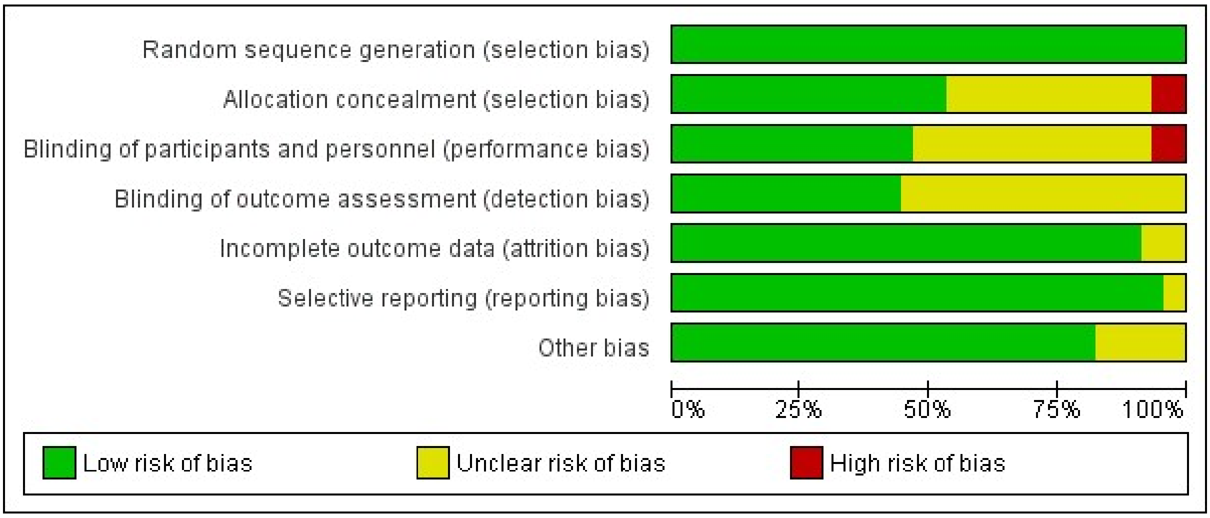

Figure S1: The risk of bias graph: succinctly illustrates the percentage evaluation of each bias item across all considered studies.

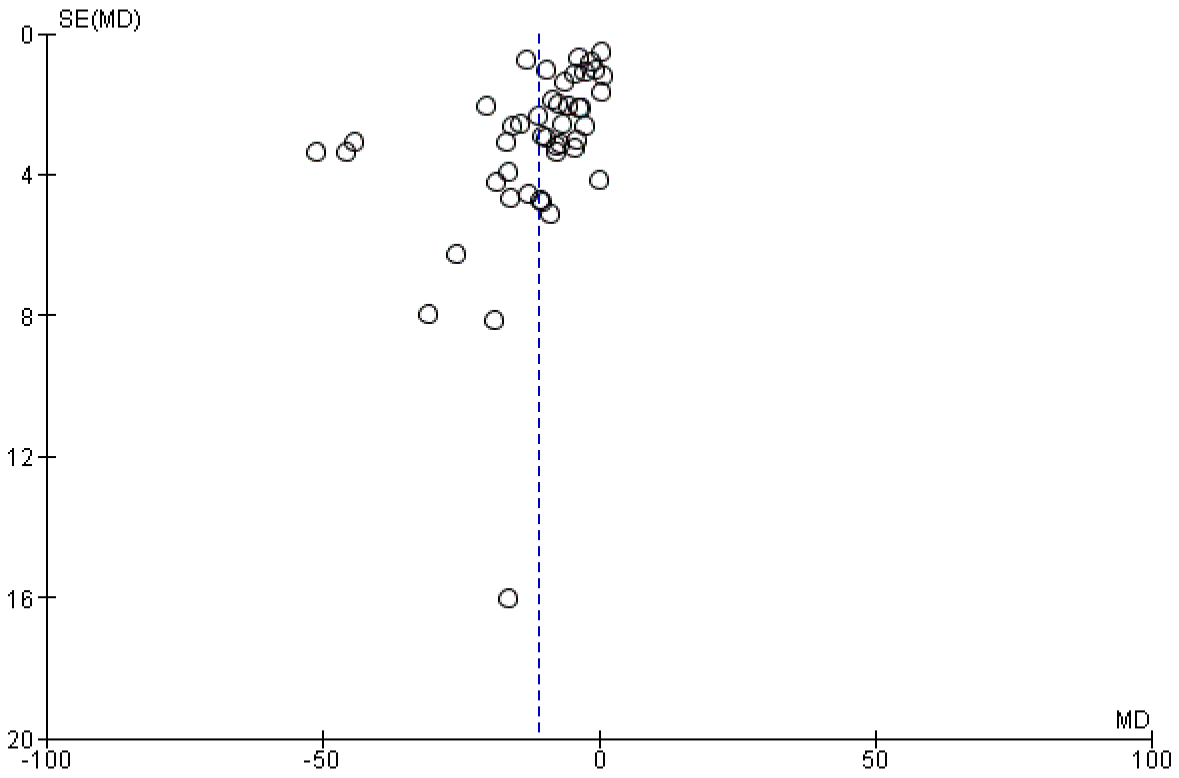

Figure S2: Funnel plot between intervention (distraction) and control

**Kendall's S statistic (P-Q)**	-386.00000
**Kendall's tau without continuity correction**	
Tau	-0.38990
z-value for tau	3.77598
P-value (1-tailed)	0.00008
P-value (2-tailed)	0.00016
**Kendall's tau with continuity correction**	
Tau	-0.38889
z-value for tau	3.76619
P-value (1-tailed)	0.00008
P-value (2-tailed)	0.00017

Figure S3: Begg and Mazumdar rank correlation

Intercept	-6.55597
Standard error	1.53709
95% lower limit (2-tailed)	-9.65580
95% upper limit (2-tailed)	-3.45614
t-value	4.26519
df	43.00000
P-value (1-tailed)	0.00005
P-value (2-tailed)	0.00011

Figure S4: Egger's regression intercept

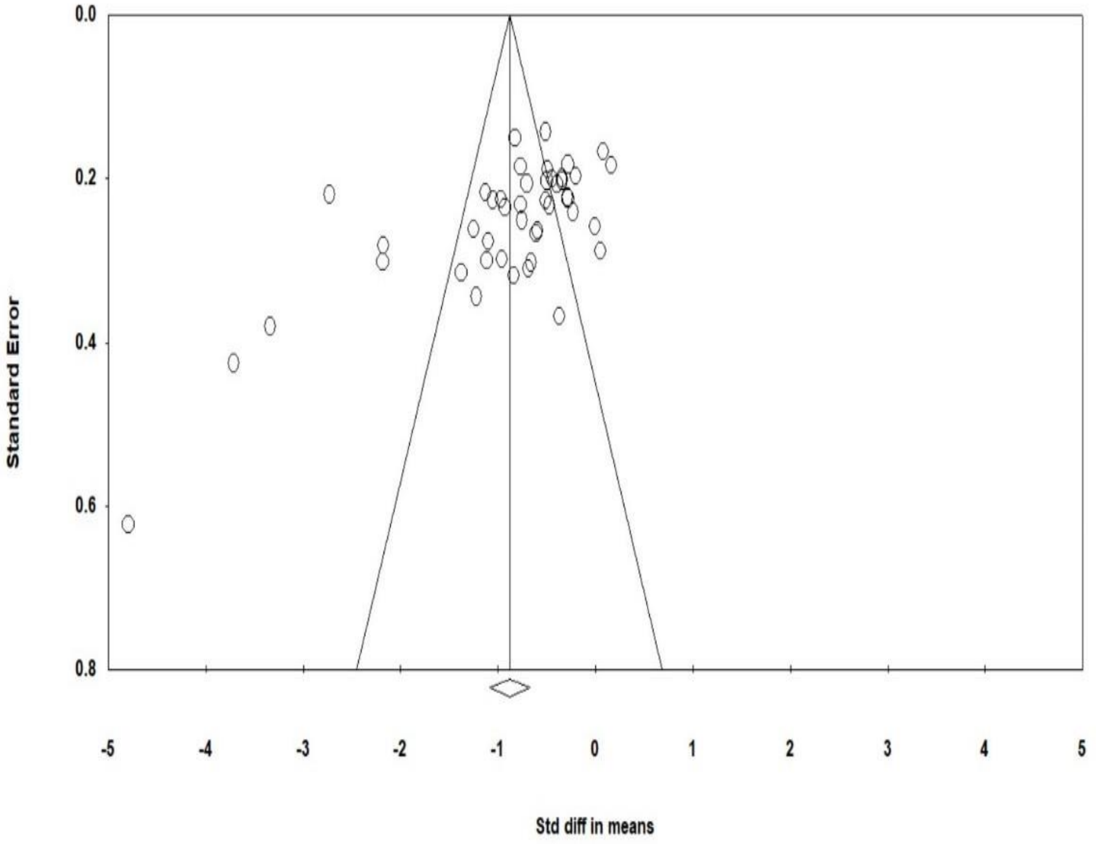

Figure S5: Funnel Plot of Standard Error by Std diff in means

		Fixed Effects			Random Effects			Q Value
	Studies Trimmed	Point Estimate	Lower Limit	Upper Limit	Point Estimate	Lower Limit	Upper Limit	
**Observed values**		-0.70272	-0.77005	-0.63539	-0.88265	-1.08956	-0.67574	401.27047
**Adjusted values**	11	-1.03011	-1.08914	-0.97108	-1.14422	-1.37226	-0.91619	802.71009

Look for missing studies where?

- ○ Not specified
- ◉ To left of mean
- ○ To right of mean

Look for missing studies using which model?

- ○ Not specified
- ○ Fixed effect model
- ◉ Random effects model

Figure S6: Duval and Tweedie’s trim and fill
